# Awake or Sleeping? Maybe Both… A Review of Sleep-Related Dissociative States

**DOI:** 10.3390/jcm12123876

**Published:** 2023-06-06

**Authors:** Maria Eduarda Sodré, Isabel Wießner, Muna Irfan, Carlos H. Schenck, Sergio A. Mota-Rolim

**Affiliations:** 1 Medical College, Potiguar University, Natal 59076-550, RN, Brazil; 2 Brain Institute, Federal University of Rio Grande do Norte, Natal 59078-970, RN, Brazil; 3 Department of Neurology, University of Minnesota, Minneapolis, MN 55455, USA

**Keywords:** dissociative states, sleep dissociation, daydreaming, lucid dreaming, false awakenings, sleep paralysis, sleepwalking, REM sleep behavior disorder, hypnosis, anesthesia, psychedelics

## Abstract

Recent studies have begun to understand sleep not only as a whole-brain process but also as a complex local phenomenon controlled by specific neurotransmitters that act in different neural networks, which is called “local sleep”. Moreover, the basic states of human consciousness—wakefulness, sleep onset (N1), light sleep (N2), deep sleep (N3), and rapid eye movement (REM) sleep—can concurrently appear, which may result in different sleep-related dissociative states. In this article, we classify these sleep-related dissociative states into physiological, pathological, and altered states of consciousness. Physiological states are daydreaming, lucid dreaming, and false awakenings. Pathological states include sleep paralysis, sleepwalking, and REM sleep behavior disorder. Altered states are hypnosis, anesthesia, and psychedelics. We review the neurophysiology and phenomenology of these sleep-related dissociative states of consciousness and update them with recent studies. We conclude that these sleep-related dissociative states have a significant basic and clinical impact since their study contributes to the understanding of consciousness and the proper treatment of neuropsychiatric diseases.

## 1. Introduction

Contemporary works on sleep regulation proposed the term “local sleep” to define physiological characteristics that can be found in specific regions of the brain during the basic states of consciousness, i.e., wakefulness, rapid eye movement (REM) sleep, and non-REM sleep (sleep onset—N1, light sleep—N2, deep sleep—N3) [1,2,3,4]. These physiological states of consciousness tend to cycle in a stable manner; however, the transitions among these states are gradual and inconsistent, with the concomitant presence of multiple state-determining biomarkers. Accordingly, this may result in mixed states in which sleep and wakefulness occur asynchronously in different regions of the brain, resulting in the so-called sleep-related dissociative states [5].

In this work, we aim to first classify these sleep-related dissociative states into physiological, pathological, and altered states of consciousness (Table 1) to then review their neurobiological substrates and related subjective experiences. By “physiological”, we mean that these states tend to occur in regular, healthy subjects, as opposed to “pathological” states that are usually associated with some disease or disturbance. Moreover, “altered” states are neither physiological nor pathological; they are exogenously induced, either by psychological or by pharmacological interventions. To illustrate these physiological, pathological, and altered sleep-related dissociative states, we describe three examples of each: (a) daydreaming, lucid dreaming, and false awakenings (physiological), (b) sleep paralysis, sleepwalking, and REM sleep behavior disorder (pathological), and (c) hypnosis, anesthesia, and psychedelics (altered).

It is important to note that the limits between these categories are fluid. For example, daydreams, which are here first considered physiological, can become pathological in specific situations, such as depression. Moreover, sleep paralysis and somnambulism are considered real diseases only if they become too frequent and/or intense. Investigating these dissociative states of consciousness is important because it contributes to furthering the understanding of human consciousness and the correct treatment of neurologic or psychiatric disturbances. In the next sections, we will describe these categories and states in detail.

## 2. Physiological States of Consciousness

### 2.1. Daydreaming

Singer and McCraven [6] defined daydreaming (or mind wandering) as a partial detachment from the reality around, directing consciousness towards the own imagination with personal content. Although this state of consciousness is part of human nature and an everyday experience, there is still little systematic knowledge about its frequency, duration, spontaneity of appearance, variations, and patterns.

Researchers have hypothesized about the advantages and importance of daydreaming, considering that it occupies more than half of the time we are awake [7]. According to these authors, daydreaming may serve for (1) Adaptive function: allowing for planning future activities, creativity, attentional cycling, and resting [8]; (2) Event simulation: there is a correlation between self-reflection, autobiographical memories, and task performance, suggesting that daydreams (as well as night dreaming) may simulate future events [9]; (3) Interpersonal relationships: allowing the development of interpersonal skills, such as compassion, moral reasoning, empathy, etc. [10]; (4) Volitional processes: one can choose to detach from the reality around to focus on one’s own thoughts [11]; (5) Personal goals: self-awareness, personal planning, goal-directed thinking, simulating another person’s perspective, and compassion [7]; (6) Mental health: daydreaming components such as imagination and fantasy support mental health, allowing the mind to varying its attentional content [7].

Singer [12] divided daydreams into three types: positive-constructive daydreaming, guilty-dysphoric daydreaming, and poor attentional control. positive-constructive daydreaming consists of carefree, positive desires and plans, such as imagining inviting a friend to dinner or being in a park with your pet. Guilty-dysphoric daydreaming is about worries and undesirable scenes, such as revenge, and undesirable experiences, such as deaths and separations. Poor attention control is not necessarily related to the emotional charge but rather an obstacle to the individual’s attention. These daydreaming experiences were studied in different ages, ethnicities, and genders [13,14].

People with more negative daydreaming, such as the guilty-dysphoric type, tend to lack volitional control over their own experience. They are more prone to less self-awareness, difficulty with concentration, and neuropsychiatric diseases such as depression and anxiety [15], as described below.

#### 2.1.1. Self-Generated Thoughts, Daydreaming, and Depression

Self-generated thoughts are intrusive imaginations, ideas, and desires that are not directly related to environmental stimuli and may arise from daydreaming [16]. Self-generated thoughts are associated with loss of reading comprehension [17], sustained attention capacity [18], and working memory [19]. Self-generated thoughts characterized by predominantly negative thoughts correlate with mental diseases, such as depression, anxiety, schizophrenia, and other pathological states of consciousness [20].

The tendency to fall into negative daydreamings, such as ruminating thoughts, correlates with depression; daydreaming can not only be related to depression but also self-generated thoughts can be a symptom of depression [21,22]. It was shown that this relationship between daydreaming and depression was more due to the type of daydreaming, that is, always ruminating on the same thoughts [18,23]. In this sense, it was found that the greater the focus and attention to the own thoughts, the greater the rumination and, consequently, the more depressed mood [23].

Marchetti, Van de Putte, and Koster [24] showed that daydreaming can predict the onset of depressive symptoms since, within the self-generated thoughts, people are faced with their own fears, desires, anxieties, and concerns, which are often accompanied by judgments that increase beliefs in more negative worldviews in relation to their own performance. According to Aldao and colleagues [25], such beliefs tend to induce depression.

#### 2.1.2. Daydreaming and the Default Mode Network

**Figure 1:**
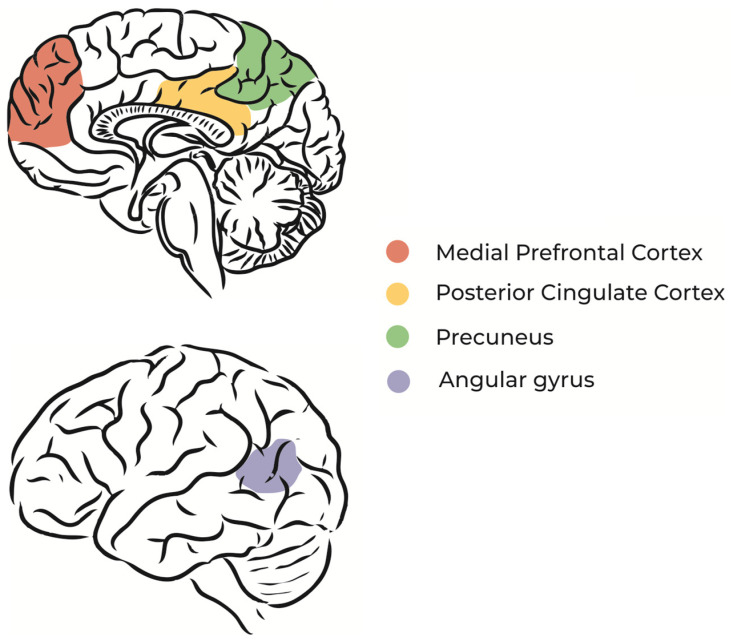
Schematic illustration of DMN main regions.

The default mode network (DMN) is a brain circuit that is activated when a person does not direct attention to environmental stimuli, during REM sleep, or during daydreaming [26,27] (Figure 1). In other words, the regions of the brain that constitute the DMN are more active in the absence of external stimuli and sustained attention [28], which means that dreaming and imagination processes share neurophysiological similarities [9].

Specific regions of the DMN, such as the dorsomedial prefrontal cortex and medial temporal lobe, are associated with thoughts about oneself (self-referential thoughts) and the construction of future scenarios based on memories [29]. Studies using functional magnetic resonance imaging during unconscious states, such as sleep and coma, found that the spatial organization of neural networks is similar to that of the resting state [30,31]. Resting-state functional magnetic resonance imaging studies show fluctuations between a positive pole that carries out activities that require attention and concentration and a negative (or default) pole that activates a specific network (DMN) during the resting state. When the DMN is activated, other networks responsible for task processing are deactivated. Concurrently, daydreaming becomes predominant, thus there is less attention span [32].

In a recent study, Hilland and colleagues [33] aimed to explore correlations between DMN dysfunction and early-onset psychosis, since there a correlation has been shown between poor DMN connectivity and schizophrenia. The analysis showed a significant reduction in DMN activity in early-onset psychosis patients compared with the healthy control group. There was no relationship between DMN activity and the use of psychotropic drugs in patients with early-onset psychosis.

#### 2.1.3. Daydreaming and Brain Rhythms

Even in tasks that require focus, shifts in the attentional focus to internal issues often occur; therefore, changes in the rhythms of the neural networks can be expected. Brabosczc and Delorme [34] analyzed the electroencephalographic signals of 12 volunteers who performed a breath-counting test and were supposed to press a button whenever they realized they had another thought occupying their minds. In these moments of concentration break, there was an increase in the frequency power of the slower theta (4–7 Hz) and delta (2–3.5 Hz) waves, whereas the frequency of the faster alpha (9–11 Hz) and beta (15–30 Hz) waves decreased. Therefore, daydreaming is associated with less vigilance and alertness, similar to sleep [35].

Supporting this idea, during daydreaming there is a performance decrease in the detection of a stimulus [14]. Since daydreaming is characterized by decreased alertness, concentration, and attention, the brain’s reaction time to external stimuli decreases. For example, a sudden change in an auditory stimulus pattern is more difficult to perceive. It was observed that the neurophysiologic reaction to this pattern change, called mismatch negativity, which is attention dependent, is smaller in the daydreaming group than in the breath-counting group. This suggests a dissociation of attention during daydreaming. In parallel, when individuals enter into a state of somnolence, the mismatch negativity is characteristic of sleep onset [36]. Subjects are absorbed in their own thoughts and personal issues and cannot maintain attention, becoming disconnected from the world [37,38].

### 2.2. Lucid Dreaming

Lucid dreaming (LD) is a specific type of dreaming characterized by the awareness that one is dreaming and the capacity to control the oneiric content [39]. LD has been mentioned in several religions and by many philosophers for thousands of years [40,41]. However, more recently, some authors were skeptical about this dream phenomenon, arguing that individuals were actually awake or had some kind of misperception [42], which might be due to the lacking evidence of LDs as genuine subjective experiences at that time.

This changed with the pioneering work of Stephen Laberge in 1980. This was based on prior research showing that: (1) eye muscles are exempt from the muscular atonia that accompanies REM sleep [43,44] and (2) direction shifts of the gaze within a dream can be accompanied by real movements of the sleeper’s eyeballs [45,46]. Laberge and colleagues thus asked lucid dreamers to move their eyes in a pre-agreed sequence (left–right movements, for example) as soon as they became lucid in the dream. This technique succeeded and laid the foundation for the scientific studies of LD [47].

#### 2.2.1. Lucid Dreaming, REM Sleep, and the Brain Rhythms

LD occurs predominantly during REM sleep [48,49]. LD has also been reported during the N1 (sleep onset) and N2 (light sleep) stages but not during the N3 stage (deep sleep) [50,51]. As for the ordinary non-LD, the LD during non-REM sleep stages 1 and 2 tends to be shorter, less vivid, less story-telling-like, and more similar to a thought or a waking memory [50] It is not clear, however, why LD is not common in the general population [52], although the majority of people show REM sleep every night. A possible explanation for this inconsistency is that LD is probably associated with specific brain rhythms during REM sleep, with different spectral characteristics from non-lucid REM sleep.

The first works observed a relationship between LD and the global alpha band power (8–12 Hz) [53,54]. Later, a study detected an increase in the beta band power (13–20 Hz) in the parietal region [55]. Subsequent works found an increased gamma band (~40 Hz) in the frontal region [56]). This observation led Voss and colleagues to propose that LD is a mixture of waking and REM sleep, which characterizes a sleep-related dissociated state [5]. A decrease in the delta band (1–4 Hz) power was also observed in the frontal and central regions [57]. Therefore, despite the numerous works reporting different spectral characteristics during LD compared with non-lucid REM sleep, there are considerable controversies regarding the brain regions and frequency bands. Possible explanations for these discrepancies are: (a) methodological aspects—limited electrode montages and different frequency bands analyses [58], (b) subjective features—different experiences and contents during LD with specific neurobiological substrates [59], and (c) proficiency levels—experienced lucid dreamers tend to have longer LD and exert more control over its content [46].

A recent work by Baird and colleagues [60] observed that LD is associated with physiological activation during REM sleep, supporting the results of previous studies of increased phasic activity during LD, including increased REM density, heart rate, respiration rate, and skin potential [48]. These findings suggest that LD correlates with increased cortical activation that peaks during phasic REM sleep. Baird et al. also observed that the increase in frontal gamma during LD [56] reflects saccadic spike potentials due to the higher eye movement density instead of heightened brain activation.

#### 2.2.2. Neuroimaging of Non-Lucid and Lucid REM Sleep

During non-lucid REM sleep, the limbic system is activated (hippocampus, amygdala, medial prefrontal cortex, and anterior cingulate cortex), which might be related to the intense emotions that are usually experienced during dreams [61,62]. On the other hand, the activity in the prefrontal cortex decreases, which may be related to the lack of self-consciousness and meta-cognition during non-lucid dreams [63,64].

Dresler and collaborators [65] observed that during LD frontal regions are activated, including the anterior prefrontal cortex, which may be associated with the insight and self-reflection abilities that characterize LD. More recently, Baird et al. [66] observed that frequent lucid dreamers showed increased resting-state functional connectivity between the anterior prefrontal cortex and other frontal, parietal, and temporal regions. Other studies suggested that LD may be associated with large-scale networks that control executive processes, such as the frontoparietal network [67]. Supporting this hypothesis, Baird et al. [66] also found that frequent lucid dreamers had increased functional connectivity between the anterior prefrontal cortex and a frontoparietal sub-network [68,69]. Another cerebral region—the temporoparietal junction—integrates visual, tactile, proprioceptive, and vestibular information, which contributes to self-consciousness and body internal imagery [70]. Overall, these findings seem to suggest that the self-consciousness and metacognitive abilities that characterize LD may be the result of a network synchronization between the frontal and temporoparietal regions. This hypothesis needs to be explored in future studies.

### 2.3. False Awakenings

False awakening (FA) is a type of dream in which people believe they have woken up since the dream content is similar to their daily life, especially regarding their activities when waking up, such as rising from bed, going to the bathroom, having breakfast, brushing the teeth, etc. Only when the subjects actually wake up do they realize they were dreaming [71]. In a study with 974 participants, 45% of them had experienced at least one episode of FA in their lifetime, 28% have it sometimes, 8% have experienced a single episode, and 7% have it often. Participants above 50 years have fewer experiences of FA (4%) than the younger ones, especially those between 21–30 years (9%) [72].

During an FA episode, the dream scenario is likely to be similar to the usual environment of the dreamer. Often, the trigger for the FAs are external factors, such as a ringing cell phone; however, sometimes even the trigger may be dreamed [71]. On the other hand, some people refer to FA as “a dream within a dream”.

#### 2.3.1. False Awakenings and REM Sleep

FA happens mostly during REM sleep, which is similar to LD, sleep paralysis (SP; see next section), and out-of-body experiences. Since they all occur in the same sleep stage (REM), these phenomena may be linked to each other. Raduga, Kuyava, and Sevcenko [72] found that people who have already experienced one of these REM sleep-dissociated states are likely to have experienced another. SP and FA share the feeling of being in a familiar environment (usually their bed), the perception of being awake, and feelings of anxiety. One main difference is the inability versus freedom to move during SP and FA episodes, respectively.

To induce FA experiences, Hearne tried to reproduce scenarios in which disturbed sleep is likely to occur, such as expectations and anxieties related to the next day. The reason for this technique is that preoccupations with upcoming events can be anticipated during dreams in the form of FA. This can occur mainly but not solely after an external trigger during sleep. Ways to identify FA depend on the person’s motivation and habit of asking him-/herself, immediately upon waking up, whether he/she was dreaming or sleeping.

#### 2.3.2. False Awakenings and the Brain Rhythms

Using polysomnography during FA, an increase in alpha frequency alternating with states of REM sleep, slow eye movement, and low muscle tone was found [73]. This study also found that, although FA and SP are experiences similar to each other and mimic a waking state, in both of them the individual is dreaming and in the REM sleep stage (associated with the predominance of theta waves in the electroencephalogram). Thus, commonalities between SP and FA regarding the characteristics of REM sleep and wakefulness can be explained by the fact that different stages of sleep or wakefulness, especially their transitions, show mixed features that characterize a sleep-related dissociated state [5]. Moreover, the nocturnal sleep of patients with SP contains a greater predominance of delta waves than in healthy individuals, suggesting an alteration in the regulation of non-REM and REM sleep [73].

## 3. Pathological States of Consciousness

### 3.1. Sleep Paralysis

Sleep paralysis (SP) is a dissociative state of consciousness that is characterized by motor, sensory, and cognitive abnormalities that usually happen in the transition from REM sleep to the waking state. It comprises the inability to move, visual hallucinations, tightness in the chest, fear of death, and perception of threatening creatures. The SP experience can induce intense frightening and anxiety due to the loss of voluntary motor activity, which can cause the inability to scream, open the eyes, cry, and even breathe [74,75]. Most people have experienced a few episodes of SP in their lifetime, but when SP occurs frequently or involves intense negative emotional components, impairing well-being, and social functioning, it can be considered a sleeping disorder (parasomnia).

Only sparsely studied by science, many cultures have interpreted SP from a supernatural perspective until today [76]. In Japan, the “kanashibari”, which can be translated as “the state of being totally bound, as if constrained by metal chains”, is caused by a vengeful spirit who suffocates his enemies [77]. In Thailand, the “phi am” is a ghost that appears when subjects are half asleep and unable to move [78]. Egyptians believe that SP is caused by the “jinn”, which are malevolent spirit-like creatures [75]. Ethiopians consider the “dukak” an evil spirit that haunts sleep [79]. In the USA, the report of “alien abductions”—experienced as the inability to move during awakening with visual hallucinations of aliens—is considered a manifestation of SP [80].

**Figure 2:**
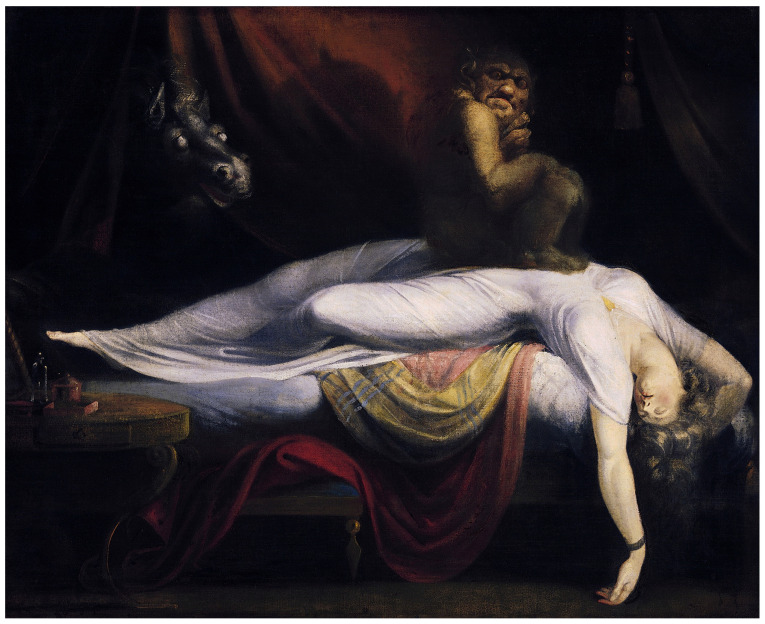
*The Nightmare* (1871), by Henry Fuseli.

The emotional components of SP have been explained by cognitive distortions such as hallucinations, which can be grouped into “Intruder”, “Unusual Bodily Experiences”, and “Incubus”. The first is characterized by the feeling of fear or the sensation of a presence in space accompanied by visual and auditory hallucinations. The second is related to out-of-body experiences. The third refers to the feeling of tightness in the chest and difficulty to breath [81], as depicted by the painter Henry Fuseli (Figure 2).

The different societies share a common frightening component due to the person’s inability to move and visual hallucinations according to the individual’s culture [82]. However, some authors interpret SP in a strictly pathological way. In the past, SP was underdiagnosed and understood as a symptom of narcolepsy. This disorder is characterized by abnormalities of sleep regulation, in which the individual falls asleep abruptly associated with cataplexy, when muscle tone is lost, usually after a very emotional episode. However, despite its similarities with narcolepsy, SP can occur separately from narcolepsy and, overall, shares few relationships with neuropsychiatric disorders. However, SP must be seen from a pathological point of view when the episodes are very frequent or intense, thus implicating physical, mental, and/or social suffering.

#### 3.1.1. Epidemiology of Sleep Paralysis

In a recent systematic review, sociodemographic and other variables related to SP were studied, including age, ethnicity, residential area, salary, breakfast consumption, alcohol consumption, substance use, stress, and trauma [83]. The authors of this systematic review found no significant age effect on the prevalence of SP. African Americans and other non-Caucasian populations showed a higher incidence of SP than Caucasians. However, other studies do not confirm this direct relationship. Among Chinese adolescents, SP was more frequent in rural than urban areas. In a study from Japan, people with higher incomes had a slightly higher incidence of SP (10.9%) than people with lower incomes (7.3%). People who eat breakfast every day had a lower incidence of SP than those who did not, although the results are inconsistent. This last association might be explained by findings that the period of food consumption may be important for the regulation of the internal circadian clock. Two large samples from China and Japan found that those who had at least one alcoholic drink the previous month were more likely (9–12%) than others (6%) to experience SP. However, in a study from the United Kingdom, this association was not found, even if related to anxiety and depressive symptoms. A history of childhood sexual abuse is strongly related to episodes of SP. Furthermore, these individuals show qualitatively more “intruder” and “incubus” hallucinations during the SP episodes. Moreover, the number of traumatic events was associated with SP cases [83].

It was reported that SP occurs more in women than men and in the supine position when sleeping. In general, at least one episode of SP during a lifetime is reported by 7.6% of the population, 28.3% among students, and 31.9% among psychiatric patients [74].

#### 3.1.2. Neurobiological Mechanisms of Sleep Paralysis

SP is related to the abrupt arrival in (or exiting from) REM sleep, when dreams occur more often and vividly. Therefore, the visual, auditory, emotional, and cognitive alterations of dreams accompany the muscle atony that characterizes REM sleep [43,84]. Similarly, brain activity during REM sleep is associated with a lack of voluntary muscle mobilization. Muscle atonia during REM sleep happens due to the presence of a descending inhibitory system from specific brainstem nuclei that projects to the anterior column of the spinal cord and then to skeletal muscles [85]. The main neurotransmitters of this complex network are GABA and glycine [86]. This intricate system prevents us from acting “in real life” the imaginary movements that we perform when dreaming, which would make us vulnerable and thus subject to being preyed upon. This notion comes from Jouvet’s work, who damaged specific brainstem nuclei and observed that animals started running, cleaning themselves, and masticating during REM sleep since they have no more muscle inhibition. These behaviors were related to dreaming [44,85]. Dahlitz and Parkes [87] consider that the mechanism responsible for bodily immobilization during SP is the lack of synchrony between changes in brain activity and muscle atonia. Thus, during SP individuals show brain activity similar to the waking period; however, the muscles are unable to move. Therefore, subjects feel awake but cannot move, a main feature of a sleep-related dissociative state [5].

Since SP is often associated with the supine body position, Jalal and Ramachandran believe that SP is linked to disorders in the parietal cortex, where monitoring of motor programs through proprioceptive feedback normally occurs. During SP, the persistent muscle atony can create a mismatch that contributes to abnormal physical perceptions and modifies the self–body image [88].

### 3.2. Sleepwalking

Sleepwalking (or somnambulism) is a condition that has been documented by humanity for a long time. In the third century AD, the Stoic philosopher Diogenes Laertius was said to read and write his works while he slept. In the second century, Galen, the “father” of anatomy, reported in his work “De motu musculorum” that he had spent a whole night walking in his sleep and only woke up after stumbling over a stone. These lines are very reminiscent of sleepwalking but they can also have alternative interpretations, especially from a poetic point of view. In the medieval period, sleepwalking and other sleep disorders were intrinsically related to religious beliefs and interpreted as divine designations or diabolical acts. The sixteenth-century Spanish writer Antonio de Torquemada wrote that the devil makes us “dream lascivious dreams” and provokes the sleepers “to commit follies for which we can lose body and mind once”. Supernatural explanations such as these still exist in many cultures [89].

Sleepwalking is a type of arousal parasomnia disorder that occurs during NREM sleep but in which the usual distinctions between wakefulness, REM, and NREM sleep are blurred, which is the precise definition of a sleep-related dissociative state. Sleepwalking is closely related to other NREM parasomnias (confusional arousals, sleep terrors, sleep-related sexual behavior, and sleep-related violence) since these sleep disturbs are mostly classified by the displayed behaviors [90]. The term disorders of arousal encompass sleepwalking, night terrors, and confusional arousals and also a broader spectrum of specific forms of NREM parasomnias, such as sexsomnia, sleep-related eating, and sleep-related asphyxia syndrome. The underlying factors associated with confusional arousal disorders are mostly unknown [91].

Sleepwalking episodes draw a lot of attention because the person is sleeping and performing complex activities, such as gesturing, pointing, walking, or even dressing, cooking, and driving. These activities can last from seconds to more than 30 min. Its main characteristics are speech in response to external stimuli, as the person is sleeping, mental confusion, variable retrograde amnesia, perceived threat, and misperception [92]. Moreover, descriptive reports show that, in children, complete amnesia is more common, as well as sleepwalking behavior characterized by automatic movements and higher arousal thresholds. Unlike children, many adults remember at least parts of the events upon waking [93]. The condition is most common in children, whereas only 2–3% of adults show sleepwalking and only 0.4% of adults sleepwalk every night. About 80% of adult sleepwalkers showed childhood sleepwalking, perhaps due to an immaturity of the sleep state [94]. Therefore, this phenomenon might be understood as a complex condition that involves high motor control levels, which we detail next.

#### 3.2.1. The Neurobiology and Pathophysiology of Sleepwalking

During the night, NREM sleep and REM sleep alternate in cycles of about 90 min. NREM sleep occurs mainly during the first half of the night’s sleep, whereas periods of REM sleep with vivid and longer dreams are predominant during the second half of the night. As said before, sleepwalking occurs especially in the deepest stage of sleep, which is the N3 sleep stage or slow wave sleep. For this reason, sleepwalking tends to occur during the first half of the night [91].

The pathophysiology of sleepwalking is not well known. Electroencephalographic studies demonstrated that sleep depth is not evenly reflected throughout the brain, with frequency differences throughout its anteroposterior axis. For example, in an epileptic patient, the motor and cingulate cortices activities differed from the associative cortical activity during the sleepwalking episode [95]. These electroencephalographic recordings showed that sleep and waking states can coexist in different regions of the brain, which characterizes a sleep-related dissociative state of consciousness [5].

In both children and adults, chronic sleepwalking is associated with other sleep disorders, which further worsen sleep quality. These include upper airway resistance syndrome, mild obstructive sleep apnea, and restless legs syndrome [96]. Therefore, sleepwalking should be seen as a sleep problem that can have negative implications for the individual’s life and thus requires specialized treatment [97].

#### 3.2.2. Treatment of Sleepwalking

Sleepwalking can bring suffering and physical dangers to the patients without their awareness, such as the risk of accidents, injuries, trauma, etc. Moreover, a study with ten sleepwalkers found that they showed daytime sleepiness even after nights with no episodes. Consequently, besides the suffering and physical dangers, this parasomnia seems to negatively impact work and overall life [98].

The main risk factors for sleepwalking are episodes that increase the need for slow-wave sleep, such as sleep deprivation or poor-quality sleep, precipitating arousal disturbances in predisposed individuals. For this reason, healthcare professionals should emphasize the importance of sufficient good-quality sleep, avoiding irregular sleep times, and having a sleep routine. `Moreover, environmental factors—such as anxiety and stress—can trigger an episode and thus should be avoided. In addition, it is important to evaluate concomitant sleep disorders that cause recurrent micro-arousals. For this, clinicians must assess and treat respiratory problems and movement disorders during sleep to control parasomnia [99]. Sleepwalking is also related to states of dissociation or confusion since it is observed in patients with psychiatric disorders and in those receiving sedatives, hypnotics, antidepressants, neuroleptics, stimulants, and antihistamines. Therefore, disorders, medications, and their combination might facilitate regional dissociation and modulate sleep and alert states leading to sleepwalking [100].

Benign episodes of sleepwalking occur occasionally and do not require pharmacological treatment. Instead, they can be addressed by changes in lifestyle, sleep hygiene, and other techniques such as psychotherapy, hypnosis (see next section), and programmed awakenings [101]. However, it is necessary to guarantee the patients’ safety if the episodes become frequent. This includes: (a) removing potentially dangerous objects from the sleeping environment, such as mirrors, glasses, and decorations, (b) locking doors and windows, and (c) sleeping on a mattress on the floor (since the subject can fall from the bed). These measures reduce the risks during the episode but do not prevent its onset [102]. These non-pharmacological measures are the first-line treatment for sleepwalking, whereas other neuropsychiatric disorders need to be excluded. Otherwise, treatment will be directed toward the underlying disease. Medical treatment for adults is only necessary if these measures are unsuccessful and the condition is associated with physical, psychological, and/or social harm [103]. However, no drug is currently approved for the treatment of sleepwalking. A few studies have been published on pharmacotherapy for sleepwalking with benzodiazepines, tricyclic antidepressants, and selective serotonin reuptake inhibitors [99].

On the other hand, many drugs can induce sleepwalking as an adverse effect. In the study by Stallman, Kohler, and White [104], the benzodiazepine receptor agonist zolpidem induced sleepwalking in a clinical trial, giving rise to numerous case reports of sleepwalking. Moreover, given the differences in individual predisposition, sleepwalking needs to be considered within the adverse risk profile, particularly for drugs that increase the GABA receptor activity, increase serotonin, or block noradrenaline activity at β-receptors [105]. Sleepwalking also needs to be considered as a possible side effect of antipsychotic drugs, although the mechanisms that may exert this effect are unknown [106].

### 3.3. REM Sleep Behavior Disorder

Rapid eye movement (REM) sleep behavior disorder (RBD) is a behavioral and experiential parasomnia that consists of abnormal behavioral release during REM sleep with loss of generalized skeletal muscle paralysis or “REM-atonia” [107]. RBD was first identified in humans in a series of five patients [108] and resembled findings from an experimental model in cats with pontine tegmental lesions [85]. Mahowald and Schenck [109] also described the dissociation and admixture of different states of consciousness that underscores the neurophysiological phenomenon in which REM-atonia is compromised and wakeful muscle tone and twitching intrudes, resulting in clinically relevant behavioral release during REM sleep. During an RBD episode, the person moves with eyes closed while attending to the inner dream action and being unaware of the actual bedside surroundings [107]. Behavioral release during REM sleep often involves acting out of dreams that are confrontational, aggressive, and violent. The enacted dreams usually involve hostile people and animals and the dreamer is often defending him/herself or loved ones against the perpetrator. The reported dream imagery closely matches the observed physical behaviors during video-polysomnography evaluation. RBD is a dream disorder almost as much as a sleep behavioral disorder. The first textbook on RBD describes various considerations in detail [110].

#### 3.3.1. Epidemiology of REM Sleep Behavior Disorder

According to a recent meta-analysis, the prevalence of idiopathic RBD is 0.68% and probable RBD was estimated at 5.65%. The prevalence was lower for European studies (3.75%) compared with American (7.94%) and Asian studies (5.39%) [111]. The typical RBD clinical profile involves middle-aged and older men with violent and injurious dream-enacting behaviors, with over 80% of these patients eventually developing an α-synuclein neurodegenerative disorder, usually Parkinson’s disease or Lewy body dementia, with an interval of about a decade from RBD onset to overt neurodegeneration [112]. A population-based study of middle-aged to older adults with polysomnographic-confirmed RBD found a 1.06% prevalence of RBD, with gender parity [113]. Younger RBD patients show greater gender parity, less severe RBD, greater association with narcolepsy-cataplexy (narcolepsy type 1), and greater association with psychiatric disorders and antidepressant use. Most antidepressant medications (especially selective serotonin reuptake inhibitors, venlafaxine, and tricyclic antidepressants) can trigger or aggravate RBD, except for the dopaminergic–noradrenergic agent bupropion [114]. Familial RBD has been reported in one family, with autosomal transmission and altered GABAergic circuits [115].

#### 3.3.2. Neurobiology and Pathophysiology of REM Sleep Behavior Disorder

RBD can manifest de novo as idiopathic RBD or can be associated with a heterogeneous group of conditions including narcolepsy, alpha-synuclein neurodegenerative disorders (especially Parkinson’s disease and dementia with Lewy bodies), paraneoplastic neurological syndromes, autoimmune disorders, central nervous system lesions (e.g., tumors, stroke), other neurological disorders, psychiatric disorders (post-traumatic stress disorders and mood disorders), antidepressant/other medications, drug withdrawal states, and toxic metabolic states.

It was demonstrated that inactivation of the sublateral dorsal nucleus in the pontine tegmentum and GABAergic/glycinergic in ventromedial medullary inhibitory neurons in murine models resulted in RBD. Neuropathological and pharmacological damage to the same nuclei in humans can lead to RBD manifestation [116,117].

#### 3.3.3. Evaluation of REM Sleep Behavior Disorder

RBD is characterized by clinically relevant motor behaviors and/or vocalizations representing dream enactment in REM sleep. The motor activity ranges from subtle upper extremity movements (often referred to as hand babbling) to more aggressive, violent, rapid jerky, and apparently purposeful oneiric behaviors such as punching, kicking, running, and jumping. These actions can be potentially injurious to patients and bed partners [107]. The frequency of dream enactment behaviors can vary from several times per night to once a month or even less. Dream recall may be inconsistent with over 40% of patients being unaware of RBD episodes, although bed partners commonly observe behaviors indicating dream enactment. RBD is the only parasomnia that requires objective video-polysomnographic confirmation to establish the diagnosis. The polysomnographic hallmark of RBD consists of electromyographic abnormalities during REM sleep called “loss of REM atonia” or “REM sleep without atonia”, with increased muscle tone and/or increased phasic muscle twitching. Other polysomnographic features include excessive tonic activity in REM sleep in chin electromyogram and/or phasic activity in REM sleep in chin or limb activity. Meticulous historical accounting and carefully monitored video-polysomnography facilitate excluding other differential diagnoses such as obstructive sleep apnea, periodic limb movements, sleepwalking, sleep talking, confusional arousals, night terrors (NREM parasomnias), and nightmares [107].

#### 3.3.4. Management of REM Sleep Behavior Disorder

The main goal of RBD management is to reduce disruptive nightmares to prevent injuries to the patient and bed partner and improve quality of life by reducing interruptions in sleep, as detailed next.

(a) Environmental modification:

Environmental accommodations to ensure safety is the mainstay of treatment (Level A evidence) according to standard guidelines [118]. Patients and bed partners should be advised to remove sharp objects, furniture, and weapons from the immediate sleeping environment to avoid injuries. Any factors that can interrupt sleep such as sleep deprivation or insufficient sleep should be addressed. Co-morbid obstructive sleep apnea should be treated to reduce arousal from the increased respiratory effort, which can be more frequent in REM sleep [119]. A pressurized bed alarm with a pre-recorded calming message in a familiar voice has been shown to help gently prompt the patient to return to sleep but is not widely used [118]. Hypnosis (see next section for more details) has been attempted to treat parasomnia including RBD with some success but is not standard practice [120]. If feasible, drugs aggravating or unmasking RBD symptoms such as selective serotonin reuptake inhibitors, serotonin and norepinephrine reuptake inhibitors, and tricyclic antidepressants should be reduced or replaced judiciously with agents that do not cause dream enactment, such as bupropion.

(b) Pharmacotherapy:

Clonazepam has been used successfully as a first-line therapy in doses of 0.5 mg to 2 mg with 90% control of nighttime behaviors (Level B evidence) [118]. Adverse effects are dose-related and include respiratory depression, somnolence, gait trouble, altered cognition, dizziness, and falls. Melatonin (dose 3–15 mg) has been increasingly used as a first-line agent (Level B evidence) due to the preferable side effect profile, especially in elderly patients with cognitive impairment [121]. A combination of both agents may be needed if single therapy is not efficacious. If these drugs fail, dopamine agonists (pramipexole, rotigotine) have also been used to treat RBD especially if periodic limb movements are also present. Other agents used are based on limited case reports and series including cholinesterase inhibitors (donepezil, rivastigmine), anticonvulsants (carbamazepine), benzodiazepines (triazolam), antipsychotics (clozapine, quetiapine), levodopa, sodium oxybate, and ramelteon.

(c) Counseling:

Patients with idiopathic RBD should be appropriately counseled about the risk of future development of alpha-synucleinopathies; this is a complex issue that has recently been comprehensively addressed [122]. Yearly surveillance should be conducted with detailed clinical evaluation for early detection and proper management of any emergent neurodegenerative disorder. This is especially relevant in our current times since the International RBD Study Group and the North American Prodromal Synucleinopathy Consortium (funded by the NIH) are enrolling idiopathic RBD patients for imminent neuroprotective trials.

## 4. Altered States of Consciousness

### 4.1. Hypnosis

The term “hypnosis” derives from the Greek Hypnos, meaning “to sleep”. It was popularized by James Braid in the middle of the 19th century to designate a sleep-like, highly suggestible state based on induction and relaxation techniques [123]. In the middle of the 20th century, Milton Erickson revolutionized hypnosis application with his individualized, non-authoritarian approaches, which laid the foundation for the development, use, and acceptance of modern clinical hypnosis [124]. Today, hypnosis is often embedded in a broader therapeutic approach and recognized as complemental treatment in psychotherapy, medicine, sports, and forensics [125]. In clinical application, it is mostly known as an efficient tool for pain reduction but might also be useful in phobias, depression, eating disorders, posttraumatic stress disorders, and substance use disorders, among many others [126].

Over two centuries of research have always strived to better describe the phenomenology, efficacy, and challenges of hypnosis; however, the technique is far from being thoroughly explored. Hypnosis can be understood as an altered state of consciousness produced by suggestions from a hypnotist to a hypnotized person to induce changes in perception, cognition, or behavior [125]. In general, the suggestions consist of three phases of: 1—induction, including general suggestions to guide the person into a hypnotic trance; 2—application, including directed suggestions around the specific topic; and 3—termination, including general suggestions to guide the person out of the trance.

#### 4.1.1. Hypnosis, Sleep, and Dreaming

Despite its name, hypnosis reflects few properties of deep sleep. Some external appearances during the hypnotic trance might resemble sleep-like behavior, such as a deeply relaxed body and behavior resembling sleepwalking when the person talks or acts, which contributed to the name of the technique [127]. Moreover, hypnotic suggestions commonly reflect sleep-related terminology, such as inducing a “deep relaxation/sleep” and terminating with “waking up”. However, these associations are rather superficial and more characteristic of the early developments of hypnosis, whereas later research has made efforts to avoid these comparisons and focus on the more central features of hypnosis. Nevertheless, hypnosis shares neurophenomenological similarities with dreaming and can interact with dreaming and sleep behavior.

Overall, hypnosis is thought to be composed of the three main mechanisms of: 1—suggestibility (i.e., non-voluntary and uncritical compliance with suggestions); 2—absorption (i.e., total perceptual, imaginative, and ideational attention); and 3—dissociation (i.e., experiences and actions detached from self-control) [128]. These may also play a role in dreaming, for example, through the spontaneous integration of external or internal stimuli (e.g., alarm clock or school exam [52]) in the dream experience (suggestibility), the total, unreflective immersion into the dream narrative (absorption), and the uncritical acceptance of bizarre dream elements (dissociation) (compare with Psychedelics Section).

Hypnosis and dreaming are characterized by an overall detachment from the external environment with an attentional focus on the current internal experience, which, in hypnosis, is externally suggested by a hypnotist [128,129]. This experience mostly consists of visual narratives but can involve all senses depending on the suggestions, similar to dreaming [64,130]). Hypnosis and dreaming are characterized by reduced self-reflective processing and loosened self-boundaries [128,130]. Both can evoke autobiographical memories but also generate false memories [131,132]. Hypnosis and dreaming also can spontaneously activate symbolic content and bizarre features [133]. Whereas meta-awareness is present in lucid but not in non-lucid dreams, it can be fostered but also inhibited in hypnosis depending on the suggestion, establishing hypnosis as compared to both dream types [67,134].

Hypnosis, dreaming, and sleep states might also be entangled and influence each other. Hypnosis can improve sleep quality [135] and be helpful in parasomnias such as nightmares, sleep terror, and sleepwalking [120]. Hypnotic dreams, i.e., suggestions to dream during the hypnotic state, can be used to induce, re-experience, intensify, and modulate nocturnal dreams or daydreams [136] and are phenomenologically similar to these [137].

Notably, associations were found between hypnotizability, i.e., the capacity to enter hypnosis, and sleep/dreaming behaviors. Hypnotizability correlated with the ease of falling asleep at night [127], the capacity to dream on a chosen topic, and the frequency of (precognitive and out-of-body) dreams [138]. Moreover, hypnotic dreams in highly hypnotizable persons resemble more nocturnal dreams than daydreams regarding length, emotionality, characters, setting, and distortions [137].

#### 4.1.2. Hypnosis and the Brain

Neurophysiologically, hypnosis has been thought to induce brain states characterized by attention, relaxation, and dissociation [139]. Numerous neuroimaging studies have examined the brain activity associated with hypnosis and found some support for these assumptions (as described below), although diverse findings beyond that have emerged. A summary of results is difficult due to the multifaceted nature of techniques, purposes, internal, and external factors, making every study unique and considerably influencing the neuronal responses. In this light, it is not astonishing that a recent meta-analysis of neuroimaging data solely reported the activation of one region correlated with hypnosis, namely the lingual gyrus [140]. This region is involved in higher-order visual perception and mental imagery, highlighting the importance of visual imagery for the hypnotic state and reinforcing subjective findings.

Beyond that, hypnosis seems to modulate three main brain networks, namely, the central executive network, salience network, and default mode network (DMN) (Figure 1), pointing to the profound impact of hypnosis on fundamental brain processes. Specifically, hypnotic depth and absorption seem to be related to changes in the dorsolateral prefrontal cortex and inferior parietal cortex (central executive network), changes in the insula and anterior cingulate cortex (salience network), and decreases in the medial prefrontal cortex and precuneus (DMN) [140]. Changes in these networks for processing task-related activities (central executive network) and self-related daydreaming (DMN), together with reductions in the processing perception of sensory networks, might underlie the subjective and behavioral findings of hypnosis-related increased attention, changed meta/body awareness, and decreased environmental processing and mind-wandering [140,141].

This notion is supported by studies investigating electroencephalographic rhythms. Here, hypnosis seems generally characterized by increased occipital gamma, a frequency related to attention and concentration in a region of visual processing, reinforcing the notion of the predominance of visual imagery in hypnosis [141]. Moreover, hypnosis was associated with increased frontal alpha and decreased central, frontal, and parietal gamma, potentially related to increased relaxation [141]. Hypnosis is also commonly associated with increased theta, a frequency related to relaxation and sleep but also to emotion and memory encoding and recall, which is why it has been speculated that theta might facilitate the response to suggestions by retrieving the corresponding perceptual/cognitive experiences from memory [142]. Notably, decreased alpha might play a role in the more specific facets of hypnosis. Precisely, subjective dissociation under hypnosis was associated with decreased frontal–parietal alpha synchrony, pointing to a role of this brain network in dissociated awareness under hypnosis [143]. Furthermore, hypnotic dreams (compared with rest hypnosis) were associated with decreased alpha, pointing to brain activations related to dream suggestions [144].

### 4.2. Anesthesia

“You will fall asleep now” is the most common sentence anesthesiologists use before administering hypnotics [145]. Trying to understand the biological basis of how the human mind shifts from an alert (or responsive) state to (natural) sleep or (artificial) anesthesia is a recent and important object of study in neuroscience. First, it is necessary to differentiate two confusing concepts: wakefulness and an “unconscious” state. In the context of anesthesia, despite the state of consciousness being defined by the behavior of the individual, the lack of response does not indicate unawareness or lack of experiences that were generated only internally, so it does not mean unconsciousness [146]. During wakefulness—the “connected” state—the contents that reach consciousness are modulated through a sensory filter, which allows the conscious perception of stimuli. However, during anesthesia—the “disconnected (or dissociated)” state—the contents of consciousness revolve around internally generated experiences, such as the individual’s imagination [147].

#### 4.2.1. Sleep, Anesthesia, and the Brain Rhythms

Sleep and general anesthesia are characterized by similar behavioral correlates, such as decreased responsiveness, arousal, and movement. In terms of brain activity, scalp electroencephalographic recordings demonstrated analogous spectral characteristics of both states, such as the presence of slow waves (~1 Hz) or delta oscillations (1–4 Hz), as observed mainly in deep sleep (N3) [145].

Modern theories of memory consolidation during sleep postulate that novel information is initially encoded in hippocampal regions and, as time passes, becomes mainly neocortical [148]. Hippocampus-dependent information is replayed during sleep, and this replay is connected to a hippocampal sharp-wave ripple (80–120 Hz), which is nested in thalamocortical spindles (12–16 Hz) and cortical slow waves (~1 Hz) [149]. Intracranial recordings during anesthesia show altered delta–alpha or delta–gamma cross-frequency coupling on the temporal scale and delta, alpha, or gamma phase synchronization on the l spatial scale [146]. These authors argue that decreased cortical network synchronization is unfavorable for information integration in large-scale networks, which is in accordance with the finding that processing in primary sensory areas is intact under general anesthesia, whereas processing in secondary sensory and higher-order association areas was reduced [150].

Recently, Zimmern [151] proposed that neural networks operate close to “criticality”, that is, at a transition between ordered and chaotic states, which could be optimal for processing and transferring information. It was observed that anesthesia renders neural activity less critical, i.e., more predictable [152]. It has been hypothesized that a high variability near the network transitions is necessary to be conscious, whereas anesthesia promotes unconsciousness by inducing a shift away from the network bifurcation [145].

#### 4.2.2. Neuroimaging Anesthesia

A recent work by Scheinin [153] tried to study the gray area between the connected and the disconnected states, such as consciousness and unconsciousness, aiming to answer the question of what happens to brain activity when conscious awareness of the world disappears. Thus, they separated volunteers into two groups: in the first, participants were awake and were randomized to receive propofol or dexmedetomidine until they stopped responding. The second group consisted of deprived wakefulness and NREM sleep. To study the transition between the levels of consciousness and the main areas of the brain affected, awakenings were applied in a forced way to capture this moment using positron emission tomography. In this way, one could observe the unresponsive state transitioning to the responsive state, followed by immediate stimuli to assess internal experiences during the preceding unresponsive condition. Functional brain imaging comparing responsive, connected versus unresponsive, and disconnected states of consciousness during constant anesthetic exposure revealed increased activity of the thalamus, cingulate cortices, and angular gyri during responsive states, indicating that these areas are fundamental for human consciousness. These structures were affected independently by the pharmacologic agent, drug concentration, and direction of change in the state of consciousness. State-specific findings were distinct and separable from the overall effects of the interventions, which included widespread depression of brain activity across cortical areas. These findings identify the central brain network for human consciousness.

### 4.3. Psychedelics

Classic serotonergic psychedelics, such as lysergic acid diethylamide (LSD), psilocybin, and ayahuasca, are gaining growing scientific attention due to their therapeutic potential in diverse psychiatric conditions, especially mood and substance use disorders [154,155,156]. After a research hiatus following political motivations by the end of the 1960s, their profound effects on the human mind are being increasingly studied [157]. Findings from the last century and modern studies highlight the fundamental effects of psychedelics on perception, cognition, and emotion, including altered perceptions of the senses, self, body, time, and space, reduced cognitive control, and heightened emotionality [158,159].

#### 4.3.1. Psychedelics and Dreaming

Psychedelics were also termed “oneirogens”, from the Greek óneiros meaning “dream” and gen “to create”, due to their phenomenological similarity to dreams regarding visual, body- and self-perception, emotionality, memory, and cognition [160]. Accordingly, psychedelics have been conceptualized as experimental dreams and dreams as hallucinatory experiences [129,161].

Regarding perception, psychedelics increase the sensitivity to and distort the perception of external and internal stimuli [158]. Dreams often induce sensorimotor hallucinations that are embedded in a narrative context and mostly detached from environmental and bodily stimuli [162]. A marked feature is a lack of awareness that one is dreaming, which is gained in LD [58,67]. Therefore, psychedelics are thought to be phenomenologically most similar to LD, a notion supported by recent findings of linguistic similarity between reports on psychedelic and dream experiences [163]. Notably, the reports on Datura plants, containing tropane alkaloids, showed linguistic similarities to non-lucid dreams, whereas sedatives, stimulants, antipsychotics, and antidepressants showed no similarity to dream reports.

On the topic of self and body, psychedelics are well known to distort, weaken, or dissolve perceived self and body boundaries, including impaired self-control, separation from and unreality of the body, and self-transcendence [164]. Dreams are mostly reflected by a self-centered perspective, although self and body boundaries might be weakened or dissolved, especially in non-lucid dreams [129], and even reflect self-transcendence [165]. In LD, body boundaries are usually strong [129], whereas the self can be divided into one experiencing and one watching part, the latter of which can influence the narrative [166].

Considering emotion and memory, dreams are characterized by high, often negative emotionality, which is thought of as the adaptive function for fear extinction through deconditioning [167]. Similarly, psychedelics intensify emotionality, including mainly positive affects but also anxiety and challenging experiences [168] and may activate autobiographical memories, memory recall, and consolidation [169,170]. Therefore, psychedelics might aid the fear memory extinction by reactivation, modulation, and reconsolidation, a notion supported by animal studies [171,172].

Regarding cognition, psychedelics decrease coherent, rational, targeted thinking, as well as attention, working memory, and cognitive control, and induce chaotic and bizarre thinking [173,174]. On the other hand, they increase associative, surprising, meaningful, and symbolic thinking [175]. Dreams are reflected by cognitive bizarreness, including incongruities, uncertainties, and discontinuities in the narrative [133] but might also facilitate cognitive flexibility, associative thinking, creativity, and insights [176,177,178,179].

Despite these similarities, there are some main differences between psychedelic and dream states, including perceived characteristic geometric patterns and a decoupled conscious perception from external stimuli under psychedelics and the lack of meta-cognition in (non-lucid) dreams. Therefore, psychedelic experiences seem to especially resemble LD [160].

#### 4.3.2. Psychedelics and the Brain

Besides phenomenology, psychedelics and dreams show neurophysiological similarities from the receptor to the brain network level. The cerebral serotonin (5-HT) receptors seem essential for mediating psychedelic effects [157]. 5-HT_2A_ receptors mediated the “dreamlike” effects of psychedelics during mental imagery [180]. 5-HT receptors also play a key role in the sleep–wake cycle and dreaming [181]. 5-HT_2_ receptors mediate some of the psychedelic effects on sleep regulation, including increased wakefulness, reduced slow-wave sleep and activity, and changed REM sleep latency, duration, and episodes [157,182,183].

Brain imaging studies reported functional changes in regions processing sensory, self, and body perception and emotion, memory, and cognition. Regarding sensory perception, psychedelics impair thalamocortical feedback loops, potentially accounting for impaired sensory filtering and inducing an information overload [158]. Moreover, they activate primary, secondary, and associative visual areas and frontal areas that partially correlate with complex imagery; in line with this, these areas are similarly activated during imagination and perception of images under psychedelics [184,185]. In dreaming, the brainstem, primary and secondary visual areas, and higher-order frontal areas are activated during the characteristic visual mental imagery. For psychedelics and dreaming, it is still a matter of debate whether these activations occur in bottom-up or top-down directions [160].

Regarding self and body perception, among the most prominently and consistently reported psychedelic effects are decreases in the default mode network (DMN) [186] (Figure 1). As said before, the DMN is considered the neural correlate of the self, daydreaming, and dreaming [27,187]. Specifically, dreams seem to deactivate the posterior cingulate cortex and precuneus [188], two DMN regions important for self-reflection and perspective-taking [189], which might explain the loosened self and body boundaries. The precuneus was increased during lucid compared with non-lucid dreaming [65]. Psychedelics activate areas processing emotion and memory, including the amygdala, hippocampus, and anterior cingulate cortex [190,191], and these areas are also activated during dreaming [61].

Regarding cognition, psychedelics decrease frontal activity and connectivity, such as in the dorsolateral prefrontal cortex, which is important for cognitive control and contextual processing, potentially accounting for less critical and controlled but more bizarre thinking styles [192,193,194]. This region is also deactivated during dreaming, which might explain the acceptance of bizarre narratives, lack of meta-awareness, and volitional control [188]. These phenomena might also be explained by reduced gamma activity, a frequency important for the integration of content from different regions and related to waking consciousness [63]. Notably, frontotemporal gamma is increased during LD [56,58] and under psychedelics [195].

Overall, these studies suggest a crucial role of the 5-HT receptors in mediating dream-like, subjective effects under psychedelics closely related to the mechanisms of dreaming. Moreover, a substantial body of evidence indicates activation of brain areas processing vision, emotion, memory, and cognition in both states, highlighting their neurophysiological similarity. Together with the abovementioned perceptual, emotional, and cognitive similarities, these findings point to neurophenomenological similarities between psychedelics and dreams, particularly LD.

## 5. Conclusions and Future Directions

In this review, we classified the sleep-related dissociative states into three categories—physiological, pathological, and altered states of consciousness—and described their neurobiology and phenomenology based on three examples each. Some of these dissociative states are directly related to sleep and dreaming, such as lucid dreaming (physiological) and REM sleep behavior disorder (pathological). However, others are more indirectly related, such as psychedelics (altered), resembling more the state of lucid dreaming. It is also important to highlight that, despite some states being classified as pathological, such as sleep paralysis and sleepwalking, they are only considered a disorder if they occur too often and/or intensely, resulting in sociological, psychological, or physical harm to the person.

For sleep research in general, the close exchange between basic and clinical investigators is crucial, since basic science can be answered by clinical questions and clinical questions can guide the direction of basic research [5]. Studying daydreams improves our understanding of depression because it is related to negative daydreaming, also known as rumination. Hypnosis is a remarkable technique that supports the treatment of many symptoms, especially pain. Since REM sleep behavior disorder is associated with Parkinson’s disease, recognizing this sleep disturbance can facilitate early diagnosis and increase treatment efficacy for patients with Parkinson’s. Future research on sleep-related dissociative states could also bring us significant results. For example, Peters and colleagues [196] recently reviewed works that investigated LD as a form of mental training of motor skills and observed that this special kind of dreaming has the potential to improve motor performance during waking, which could be a revolution in sports science. Moreover, psychedelics such as ayahuasca have shown promising results for treatment-resistant depression and are now being tested in other psychiatric syndromes.

Beyond this collaboration between basic and clinical research, to fully understand the different physiological, pathological, and altered sleep-related dissociative states, anthropological, historical, and philosophical approaches are equally necessary. The collaboration of biomedical and social sciences can be extremely fruitful for future studies that aim to investigate the nature of human consciousness, as well as for developing new treatments for neuropsychiatric diseases.

## Figures and Tables

**Table 1:** Sleep-related, dissociative states of consciousness.

Physiological	Altered	Pathological
Daydreaming	Hypnosis	Sleep paralysis
Lucid dreaming	Anesthesia	Sleepwalking
False awakening	Psychedelics	REM behavior disorder

## Data Availability

This is a review article, thus there is no data to share.
